# The Palliative and Antioxidant Effects of Hesperidin against Lead-Acetate-Induced Testicular Injury in Male Wistar Rats

**DOI:** 10.3390/biomedicines11092390

**Published:** 2023-08-26

**Authors:** Rasha Abu-Khudir, Hayfa Habes Almutairi, Sahar S. Abd El-Rahman, Karim Samy El-Said

**Affiliations:** 1 Chemistry Department, College of Science, King Faisal University, Al-Ahsa, P.O. Box 380, Hofuf 31982, Saudi Arabia; halmutairi@kfu.edu.sa; 2 Chemistry Department, Biochemistry Branch, Faculty of Science, Tanta University, Tanta 31527, Egypt; kareem.ali@science.tanta.edu.eg; 3 Department of Pathology, Faculty of Veterinary Medicine, Cairo University, Giza 12211, Egypt; saharsamirmah1@cu.edu.eg

**Keywords:** heavy metals, oxidative stress, testicular toxicity, male infertility, natural antioxidants

## Abstract

Lead (Pb)-induced reprotoxicity is a detrimental consequence of Pb exposure, which results in abnormal spermatogenesis, testicular degeneration, and pathogenic sperm changes. The association between impaired male reproductive function and Pb-induced oxidative stress (OS) has been demonstrated, with consequent testicular antioxidant deficiency. The current study investigated the protective role of the natural antioxidant hesperidin (HSD) against lead-acetate (PbAc)-induced testicular toxicity. Male Wistar rats (n = 40) were randomly divided into four experimental groups: Group I (negative control) received 2.0 mL/kg BW 0.9% saline; Group II received 100 mg/kg BW PbAc; Group III received 100 mg/kg BW HSD; and Group IV received HSD two hours before PbAc using the abovementioned doses. The treatments were administered daily for 30 consecutive days. The results showed that HSD treatment significantly restored PbAc-induced decrease in body, epididymal, and testicular weights as well as in semen parameters, reproductive hormones, and testicular markers of OS. Reduced MDA levels and improved testicular histopathological findings were also observed. Collectively, this study sheds light on the preventive role of HSD against PbAc-induced testicular injury, which is mediated via the suppression of OS and the modulation of reproductive hormones as well as the plausibility of HSD being used as a supplementary therapeutic option for recovery.

## 1. Introduction

Approximately 50% of overall infertility cases in 10–15% of infertile couples are attributed to male infertility factors [1,2]. Numerous conditions, including systemic illnesses, endocrine abnormalities, obesity, malnutrition, genetics, and environmental hazards can lead to impaired spermatogenesis and eventually male infertility [3,4,5,6].

According to epidemiological and animal studies, exposure to heavy metals could adversely affect male fertility either directly via impairing gonadal structures or indirectly through disruption of endocrine functions [7,8,9]. The risk of exposure to heavy metals has increased due to rising environmental pollutants and due to their non-biodegradability [10,11].

Heavy metals can induce oxidative stress (OS) via the increased production of reactive oxygen species (ROS) and decreased antioxidant capacity. Consequently, oxidative-stress-induced damage in testicular tissue occurs, which can result in poor semen quality and infertility [12,13,14]. Namely, the hazardous metal cadmium (Cd) has been reported to induce mammalian testicular damage. In addition to loss of spermatozoa, alteration of the blood–testis barrier, Sertoli cells (SCs), and seminiferous tubules are well-established effects of Cd. Moreover, Cd causes alteration of the development and function of Leydig cells as well as disruption of the vasculature of testes. Previous studies have shown that Cd-induced generation of oxidative stress in testicular tissue is one of the major mechanisms involved in Cd toxicity that result in male infertility in different species [15,16]. In addition to Cd, adverse effects of mercury (Hg) on male reproductive functions in various experimental animals have been previously reported. The most significant testicular changes resulting from exposure to Hg include, among others, impaired spermatogenesis, sperm pathologies, and testicular atrophy [17]. Furthermore, chromium (Cr) is another widely dispersed heavy metal in the environment [18]. Hexavalent chromium, Cr(VI), the most toxic form of Cr, is broadly known for having adverse effects on male reproduction [19]. The reprotoxic effects of Cr(VI) in mammals and several marine invertebrates have been previously reported [20,21].

Among heavy metals, lead (Pb) is one of the most pervasive and dangerous environmental toxins. The most common sources of lead exposure implicated in severe injuries to vital organs include lead-based paints from older buildings, lead-contaminated water, gasoline, coal combustion, batteries, electronic wastes, cosmetics, and traditional medicines [22,23,24]. Lead toxicity is a significant environmental health problem with damaging effects on the human body, represented by various clinical manifestations that are dependent upon the absorbed dose as well as the route and duration of exposure [25,26].

Regarding Pb-induced reprotoxic effects, exposure to Pb adversely affects semen and sperm quality, though the underlying mechanisms are still relatively unclear. Several studies revealed a substantial inverse relationship between elevated Pb levels and common semen parameters and biomarkers of sperm function [27,28]. A close relationship between impairment of male reproductive function and Pb-induced OS with consequent antioxidant depression has been previously reported. Moreover, inhibition of steroidogenesis in Leydig cells can occur due to high ROS levels [29,30,31].

In addition to impaired semen and sperm quality resulting from Pb-induced OS, Pb is regarded as a potential endocrine disruptor in exposed individuals; it affects semen quality and male fertility via alteration of hormonal production and regulation [32,33,34,35]. For instance, previous animal studies reported an association between Pb-induced reprotoxicity and a significant reduction in serum levels of the pituitary gonadotropins—follicle-stimulating hormone (FSH) and luteinizing hormone (LH), as well as testosterone (T) [36,37].

Antioxidants, either endogenous (naturally produced in the human body) or exogenous (supplied externally through diet and/or supplements), are used by our bodies to counteract OS [38,39]. In fact, sperms are believed to be more vulnerable to OS compared to other cells due to diminished cytoplasmic content, reduced concentration of ROS-suppressing antioxidants, as well as high concentration of polyunsaturated fatty acids [40,41,42]. Hence, the presence of antioxidants in sperm cells is essential for their protection against oxidative stress-induced damage and is also necessary for the modifications occurring in sperm maturation [43,44]. Several studies have highlighted the advantages of antioxidant supplementation for the treatment of oxidative stress-induced male infertility, where the resulting DNA damage was significantly reduced and sperm parameters were remarkably improved [45,46,47]. Notably, several previous in vivo studies have pointed to the protective effects of natural nutraceutical antioxidants, including plant derived flavonoids, against cadmium-induced testicular OS, inflammation, apoptosis and/or autophagy [48,49,50]. Similar protective effects of natural flavonoids have been reported against testicular toxicity induced by other heavy metals [51,52,53,54,55]. Moreover, naturally occurring flavonoids, including citrus flavanones, are recognized for their positive effects on human health, including the reduction in risks associated with exposure to environmental contaminants. In various pre-clinical and clinical trials, flavonoids appeared to counteract the impairment of male fertility and gonadal development, as well as the advancement of cancers of the reproductive systems of both sexes, which are caused by exposure to organic and inorganic contaminants [56].

Hesperidin (HSD; 3′,5,7-trihydroxy 4′-methoxyflavanone 7-rutinoside, hesperetin 7-rutinoside) is a flavanone glycoside found abundantly in citrus fruits (family *Rutaceae*) [57,58] and is a prominent natural antioxidant known for its ability to reduce OS [59,60]. Moreover, numerous pharmacological activities of HSD have been reported, including, among others, anti-inflammatory, anticarcinogenic, and antimicrobial activities [61,62,63]. Notably, the pharmacological applications of HSD relied on its various advantages, including high safety profile, non-accumulative nature, and few adverse effects [64,65,66,67]. Remarkably, several studies have demonstrated the protective effects of HSD, based on its antioxidant and free radical scavenging properties, against testicular toxicity in various animal models [68,69,70,71]. Although the protective properties of some naturally occurring antioxidants against Pb-induced testicular toxicity in animals have been previously investigated [72,73,74,75], that of HSD has not been addressed yet. Hence, the non-toxic nature of HSD and its antioxidant effect have prompted us to unravel its protective role against Pb-induced testicular toxicity at both the biochemical and histopathological levels.

## 2. Materials and Methods

### 2.1. Chemicals and Kits

Lead acetate (PbAc trihydrtae; Cat. No. 32307) and hesperidin (HSD; Cat. No. H5254) were purchased from Sigma-Aldrich (St. Louis, MO, USA). Mouse/Rat testosterone (T) ELISA kit (Cat. No. EK7014) was purchased from Boster Bio (Pleasanton, CA, USA). Rodent follicle-stimulating hormone (FSH; Cat. No. KA2330) and luteinizing hormone (LH; Cat. No. KA2332) ELISA kits were purchased from Abnova (Taipei, Taiwan). Assay kits for determining reduced glutathione (GSH; Cat. No. GR 2511), catalase (CAT; Cat. No. CA 2517), superoxide dismutase (SOD; Cat. No. SD 2521), and malondialdehyde (MDA; Cat. No. MD 2529) were purchased from Bio Diagnostic (Dokki, Giza, Egypt).

### 2.2. Animals and Experimental Design

Forty adult male Wistar rats (weighing 150 ± 5 g) were obtained from Nile Pharma Company (Cairo, Egypt). The rats were housed under laboratory standard experimental conditions (temperature 23 ± 2 °C, relative humidity 55 ± 5%, balanced diet, and free access to water) and were acclimatized to such conditions for a period of 1 week before starting the experiment. All animal experiments were carried out according to relevant guidelines and regulations for experimental animals’ uses in research and approved by the Research Ethical Committee at the Faculty of Science, Tanta University, Egypt; approval number: IACUC-SCI-TU-0294.

**Figure 1:**
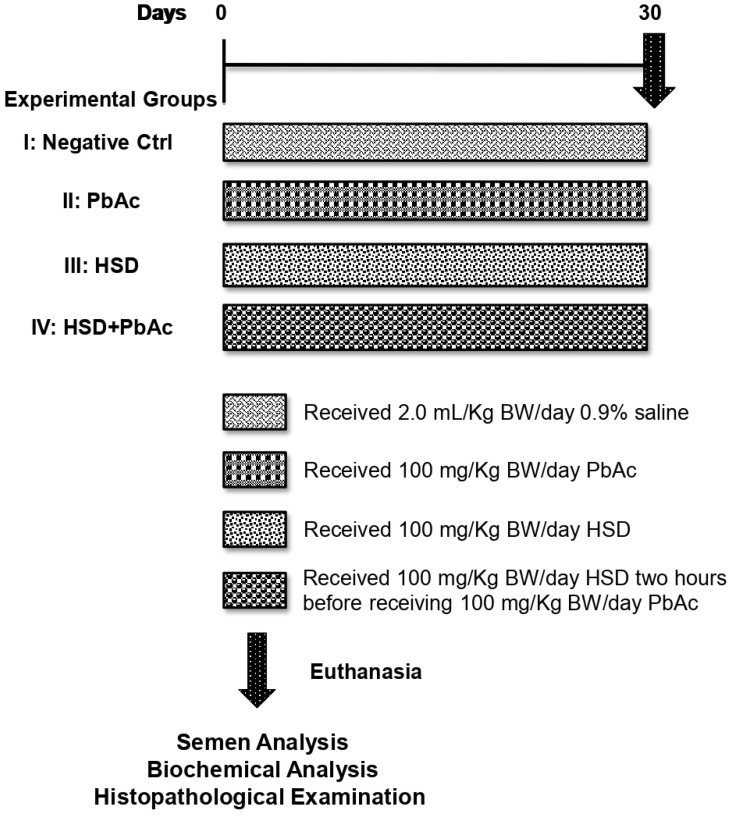
Schematic illustration of experimental design.

The experimental protocol followed in the current study is illustrated in Figure 1. Male rats were assigned randomly into four groups (n = 10/each) as follows: Group I (negative control group) orally received 0.9% saline solution. Group II (PbAc group) received 100 mg/kg BW PbAc via orogastric (OG) intubation based on previously reported significant decrease in sperm count and marked testicular histopathological changes [76]. Group III (HSD group) orally received 100 mg/kg BW HSD according to previously reported antioxidant activity [69,77]. Group IV (HSD+PbAc group) received HSD two hours before PbAc, as above indicated. Treatments were given once daily for 30 consecutive days in addition to a standard pelleted diet and water ad libitum. Body weights (BW) were recorded weakly and changes in the percentage of BW (% BW) were calculated in relation to the initial BW. All treatments were adjusted according to the rat’s body weight changes.

### 2.3. Semen Analysis

Upon termination of treatment, the rats were anesthetized using isoflurane for euthanasia. Following euthanasia, epididymal and testicular tissues were immediately excised and weighed. For semen analysis, the excised cauda epididymis was trimmed of fat, finely minced in physiological saline, and allowed to incubate at 37 °C for dispersion of sperms. Epididymal sperm count was carried out using a Neubauer’s counting chamber (hemocytometer), where the diluted sperm suspension was transferred to each counting chamber, allowed to stand for 5 min, and thereafter observed under the light microscope at 40× magnification. For sperm motility assessment, the percentage of forward progressive sperm motility was estimated visually using the light microscope at 400× magnification. The proportions of sperms moving forward and those that did not move were considered motile and non-motile, respectively. Afterward, the percentages of motile sperms were determined as previously described [78]. Sperm viability was determined using the eosin stain method based on the dye exclusion principle. The percentage of live (viable) and dead (non-viable) sperms was estimated by mixing one drop of freshly collected semen and two drops of eosin solution for 1–2 min. The mixture was smeared on a clean microscope slide, allowed to air dry, and evaluated via light microscopy. Accordingly, non-vital sperms with damaged plasma membranes were stained, whereas vital sperms with intact cell membranes were not. The percentage of dead sperms defined sperm viability [78]. Sperm morphological abnormalities in a total of 400 sperms were assessed as previously described [79]. Briefly, a drop of the prepared sperm suspension was smeared on a pre-warmed microscope slide and stained with Wells and Awa’s stain. After being air-dried, stained smears were examined for morphological abnormalities under the light microscope. Sperms with head or tail defects were considered abnormal.

### 2.4. Assessment of Oxidative Stress Biomarkers in Testicular Tissues

Tissues of the excised right testes were used for the preparation of tissue homogenate in ice-cold 0.1 M phosphate buffer (pH 7.4) with 150 mM KCl and centrifuged at 10,000 rpm for 10 min at 4 °C using benchtop refrigerated centrifuge (Sigma 3-18KS 10370; Sigma-Laborzentrifugen, Osterode am Harz, Germany). The supernatant obtained was used for the estimation of enzymatic (CAT and SOD) and non-enzymatic (GSH) antioxidants, as well as the biomarker of lipid peroxidation (LPO), MDA, using corresponding enzyme-linked immunosorbent assay (ELISA) kits according to the manufacturer’s instructions and guidelines. Protein content in testicular tissue homogenates, used to calculate enzymatic antioxidant content, was estimated as previously described [80], using bovine serum albumin (BSA) as the standard.

### 2.5. Blood Sampling and Hormonal Analysis

Blood was withdrawn via cardiac puncture from anaesthetised rats via cardiac puncture prior to immediate euthanasia. Samples were collected into Wasserman tubes and allowed to clot at room temperature for 30 min. Serum samples were separated via centrifugation at 3000 rpm for 15 min (Laboratory centrifuge CD-0412-50; PHOENIX Instrument GmbH, Garbsen, Germany), transferred into clean dry tubes, and kept frozen in aliquots at −20 °C until needed for hormonal analyses. Collected sera were subjected to the determination of testosterone (T), FSH, and LH levels using the corresponding enzyme-linked immunosorbent assay (ELISA) kits, according to the manufacturer’s instructions and guidelines.

### 2.6. Histopathological Examination

Tissues of the left testes were fixed in 10% buffered formalin for 48 h in preparation for histopathological examination. Testicular tissues that had been fixed were dehydrated in ethanol concentrations of 30, 50, 75, 95, and 100% before being cleared in xylene and being embedded in paraffin wax. Using a sledge microtome, 5 µm thick paraffin slices were created. The obtained sections were routinely deparaffinized and stained with hematoxylin and eosin (H&E) for light microscopy [81].

### 2.7. Statistical Analysis

Statistical analysis was performed using one-way analysis of variance (ANOVA) followed by Tukey’s multiple comparisons post hoc test using GraphPad Prism 8 software package. For changes in initial and final body weights among experimental groups, two-way analysis of variance followed by Sidak’s post hoc was used. Data were presented as mean ± SD (n = 8 rats/group) with an acceptable level of significance of *p* ≤ 0.05.

## 3. Results

### 3.1. Effect of Treatment with HSD and/or PbAc on Body Weight (BW) Gain, as Well as Epididymal and Testicular Weights

**Figure 2:**
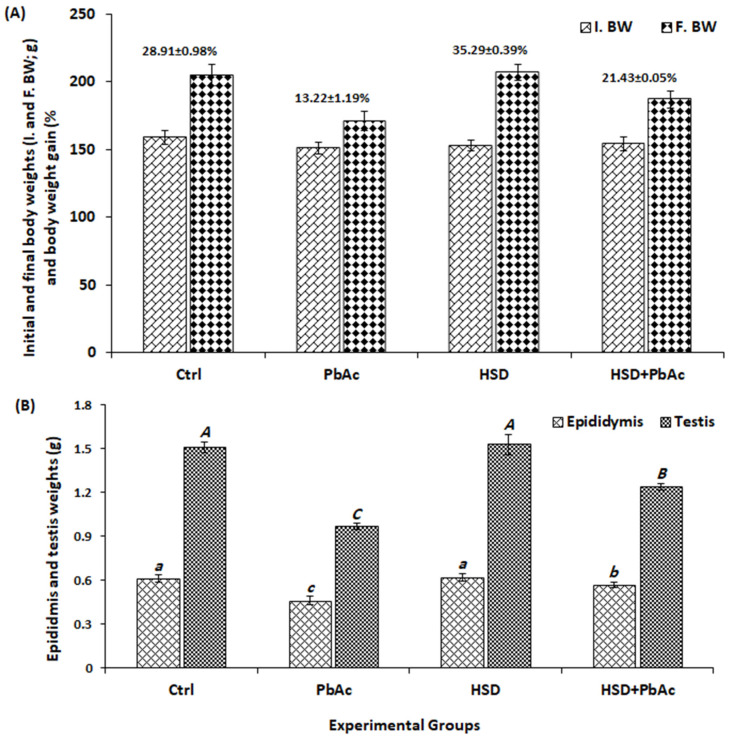
(**A**) The initial (I. BW) and final (F. BW) body weights and the percentage of body weight gain among experimental groups. (**B**) The epididymal and testicular weights among experimental groups. PbAc: lead acetate; HSD: hesperidin. Lowercase and uppercase letters represent significant differences in epididymal and testicular weights among experimental groups, respectively. Different letters on bars indicate significant difference at *p* ≤ 0.05.

The obtained results showed a highly significant (*p* < 0.001) increase in final body weights (F. BW) compared to the initial ones (I. BW) among all experimental groups. Furthermore, the percentage (%) of body weight gain in the PbAc group (13.22 ± 1.19%) showed a highly significant decrease (*p* < 0.001) compared to all the other experimental groups and resulted in approximately 15.7, 22.1, and 8.21% loss of body weight gain compared to the control, HSD, and HSD+PbAc groups, respectively (Figure 2A). In the PbAc group, the epididymal and testicular weights exhibited a highly significant decrease when compared to the control and HSD groups. Pre-treatment of the PbAc-intoxicated animals with HSD (HSD+PbAc group) led to a significant improvement in the epididymal and testicular weights when compared to the PbAc group (Figure 2B).

### 3.2. Effect of Treatment with HSD and/or PbAc on Semen Analysis

As shown in Table 1, sperm count was significantly decreased (*p* < 0.001) in the PbAc- group when compared to the control group. However, a highly significant increase was observed in the HSD+PbAc group. A comparable significant decrease in motility and viability percentages was observed among the PbAc group when compared to the control one. On the other hand, pre-treatment with HSD of PbAc-intoxicated rats resulted in a highly significant increase (*p* < 0.001) in the HSD+PbAc group. Furthermore, animals that were intoxicated with PbAc exhibited a highly significant increase (*p* < 0.001) in the percentage of abnormalities compared to the control ones. Nevertheless, an improvement in such an attribute was attained via pre-treatment with HSD (HSD+PbAc group) to levels showing no significant difference compared to those in the control and HSD ones. Morphological analyses of the spermatozoa revealed that the percentage of abnormalities was more noticeable in the head rather than in the mid piece and tail.

### 3.3. Effect of Treatment with HSD and/or PbAc on Testicular Oxidative Stress (OS) Biomarkers

The activity of the testicular enzymatic antioxidants CAT and SOD in the PbAc group exhibited a highly significant decrease (*p* < 0.001) when compared to the control and HSD groups of animals. However, pre-treatment of PbAc-intoxicated rats with HSD (HSD+PbAC group) led to a highly significant increase (*p* < 0.001) in CAT activity, while a significant increase (*p* < 0.05) in SOD was observed when compared to the PbAc group as shown in Table 2.

In addition to the abovementioned enzymatic antioxidants, the level of the non-enzymatic antioxidant GSH was also assessed. Compared to the control and HSD groups, the PbAc-intoxicated rats showed a highly significant (*p* < 0.001) decrease in their GSH levels. Pre-treatment of PbAc-intoxicated rats with HSD resulted in a highly significant improvement in this reduction as was observed in the HSD+PbAc group (Table 2).

As shown in Table 2, exposure to PbAc resulted in a highly significant increase (*p* < 0.001) in the testicular tissue level of MDA compared to the control and HSD groups, whereas a highly significant decrease (*p* < 0.001) in MDA level was observed in PbAc-intoxicated animals pre-treated with HSD (HSD+PbAc group).

Collectively, these findings suggest that pre-treatment with HSD can increase the levels of testicular enzymatic and non-enzymatic antioxidants and decrease LPO in PbAc-intoxicated male Wistar rats.

### 3.4. Effect of Treatment with HSD and/or PbAc on Serum Levels of Follicle-Stimulating Hormone (FSH), Luteinizing Hormone (LH), and Testosterone (T)

**Figure 3:**
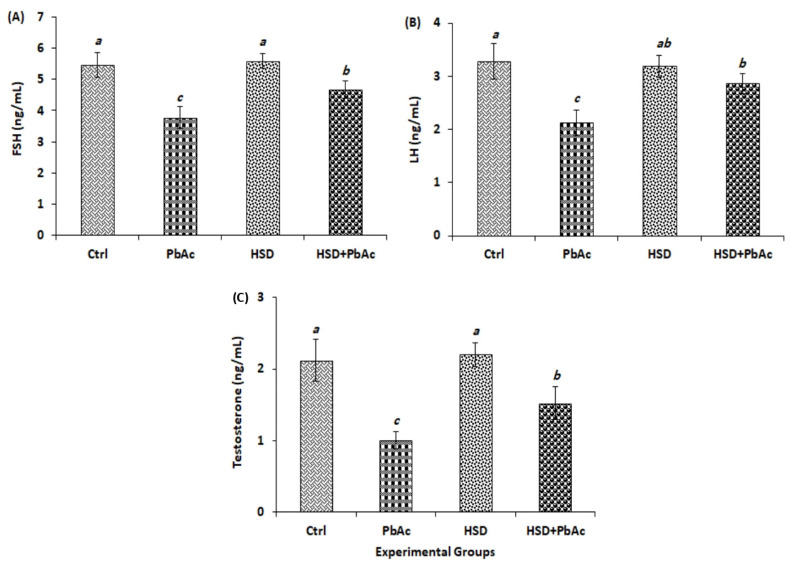
Serum levels of (**A**) follicle-stimulating hormone (FSH), (**B**) luteinizing hormone (LH), and (**C**) testosterone among experimental groups. PbAc: lead acetate; HSD: hesperidin. Different small letters on bars indicate significant difference at *p* ≤ 0.05.

As compared to the control and HSD groups, a highly significant decrease (*p* < 0.001) in the serum levels of FSH, LH, and T was observed among the PbAc group. However, the pre-treatment of PbAc-intoxicated rats with HSD led to a highly significant increase (*p* < 0.001) in the serum levels of FSH, LH, and T in the HSD+PbAc group compared to the PbAc group (Figure 3).

### 3.5. Effect of Treatment with HSD and/or PbAc on Testicular Histology

**Figure 4:**
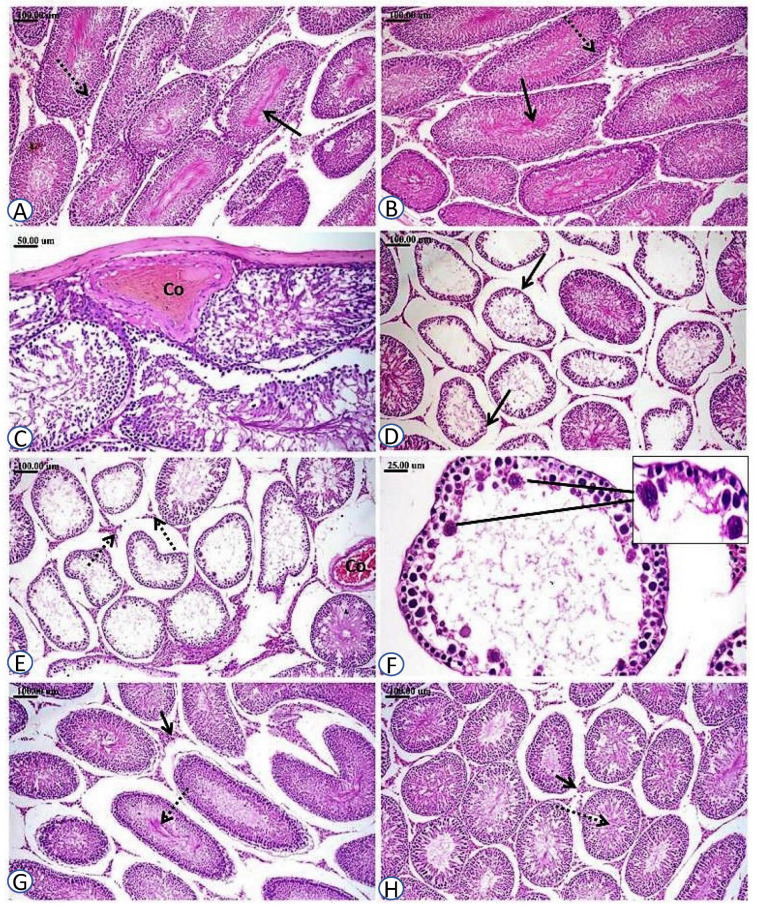
Photomicrographs of testicular sections of male Wistar rats from all experimental groups. (**A**,**B**) Sections from control and HSD groups revealed normal histological structure with normal luminal sperms (solid arrow) and spermatogonial layers (dotted arrow); (**C**–**F**) sections from PbAc group with marked alterations of testicular tissue with congestion of testicular vessels (Co) and defective spermatogenesis (solid arrow), as well as Leydig cells degeneration (dotted arrow), scattered necrosis, and spermatid giant cell formation (upper insert); (**G**,**H**) sections from HSD+PbAc group showed a positive protective effect of HSD on testicular histology, resulting in sustained integrity of spermatogoneal cells (solid arrow), normal Leydig cells (short solid arrow), and active spermatogenesis (dotted arrow).

In the present study, histopathological examination of testicular tissues excised from the control and HSD groups revealed normal histological structures (Figure 4A,B, respectively). As shown in the figures, the seminiferous tubules appeared lined by spermatogoneal cells in various stages of development and Sertoli cells (SCs) with active sperms in their lumens, as well as Leydig cells in the interstitial tissue among the seminiferous tubules. On the other hand, PbAc-intoxicated rats showed marked alterations of testicular tissue, where congestion of the testicular vessels and defective spermatogenesis were prominent findings (Figure 4C). Many seminiferous tubules showed loss of spermatogenic series, nuclear pyknosis, and severe degenerative and necrotic changes in the spermatogoneal cells without any evidence of spermatogenesis in these tubules (Figure 4D,E). Scattered seminiferous tubules appeared as only lined by SCs with prominent spermatid giant cells in their lumens, and Leydig cell degeneration and scattered necrosis were also noticed (Figure 4F), whereas pre-treatment of the PbAc group animals with HSD showed a marked positive protective effect on testicular histology, where both the spermatogoneal cells’ integrity and active spermatogenesis were sustained and normal Leydig cells were observed (Figure 4G,H).

## 4. Discussion

Given their pervasive environmental presence, exposure to heavy metals, among other environmental toxicants, can result in testicular injury and sex hormone disturbances that exhibit a negative impact on male fertility [82,83]. Among heavy metals, exposure of humans and experimental animals to lead (Pb^2+^) resulted in lowered semen and sperm quality, inhibition of steroidogenesis in Leydig cells due to high levels of ROS, as well as changes in hormonal synthesis and regulation [35,36,37,84,85]. Hesperidin (HSD) is a natural antioxidant that has a wide range of pharmacological effects [62,63]. It has been suggested that HSD can counteract the molecular changes and toxicities brought on by toxic heavy metals via antioxidant, anti-inflammatory, and anti-apoptotic effects [86,87,88,89]. Moreover, HSD exhibited a protective effect against testicular damage in a variety of animal models [68,70,71]. However, there is still a lack of scientific knowledge regarding the impact of HSD on lead-induced testicular dysfunctions in rats. Hence, the current study sought to evaluate the potential repro-protective effect of HSD against testicular toxicity resulting from PbAc-induced OS in male Wistar rats using biochemical and histological investigations.

In the current study, male Wistar rats exposed to PbAc (PbAc group) exhibited a highly significant decrease in body weight gain compared to all the other experimental groups. The loss of body weight associated with exposure to PbAc may be attributed to Pb-induced decreased food intake, increased catabolic state, and alterations in zinc-dependent enzymes resulting in disrupted nutrient metabolism [90,91]. Another reason behind the observed loss of body weight might be the reduced muscle mass and cachexia resulting from PbAC-induced OS [92,93]. Our results are consistent with other studies, which show that Pb toxicity is clearly associated with a decrease in the body weight of exposed subjects [85,94,95]. Notably, a significant increase in body weight was observed in the HSD and HSD+PbAc groups that could be attributed to the antioxidant activity of HSD [96].

In addition to body weight, a significant reduction in epididymal and testicular weights in the PbAc group was observed compared to the control and HSD groups. Similarly, reductions in epididymal and testicular weights have been formerly reported consequent to PbAc exposure [75,97,98,99,100]. The observed reduction in epididymal weight might be due to decreased sperm count [75,97,101]. Moreover, the observed reduction in the testicular weight may be attributed to direct effects of PbAc, including Pb deposition and subsequent OS, which result in testicular parenchymal atrophy, various deteriorating histological abnormalities, as well as inhibition of spermatogenesis [37,97,102]. Furthermore, it could be attributed to indirect effects of PbAc on the hypothalamic–pituitary–testicular (HPT) axis, resulting in reduced testosterone levels, where adequate bioavailability of testosterone is crucial for the structural and functional integrity of male reproductive organs [37,75,101,103]. Despite these findings, a significant restoration of both tissues’ weights has been achieved upon HSD pre-treatment of PbAc-intoxicated rats. Such a finding could be explained by the capability of HSD to increase sperm count and to reduce Pb-induced testicular histological damage, which are reported in the current study.

Toxic heavy metals exert a deleterious impact on reproduction and are closely correlated to reduced semen quality [3,31,104]. A growing body of evidence suggests that sperm quality is the most significant predictor of male fertility [105,106,107]. In the present study, exposure to PbAc resulted in a highly significant decrease in sperm count, motility, and viability compared to all the other investigated experimental groups, as well as a highly significant increase in sperm morphological abnormalities. Similar impaired semen parameters were previously reported in experimental animals exposed to PbAc [85,108,109]. Such an impairment might be attributed to the direct toxic effects of PbAc on sperm cells as well the enhanced production of ROS [31]. Moreover, histopathological findings have previously revealed the adverse effects resulting from Pb exposure on male accessory glands, which play a crucial role in the normal physiology of sperms [110,111,112]. However, the pre-treatment of PbAc-intoxicated animals with HSD resulted in significant improvement in the impaired seminal parameters caused by PbAc exposure.

Testicular OS has long been recognized as a key factor in male infertility [113,114]. At physiological levels, ROS are actively engaged in the control of spermatogenesis and fertilization [115]. However, the overproduction of ROS triggers OS in spermatozoa by reacting with polyunsaturated fatty acids (PUFA), which are abundant in spermatozoal lipid membranes. This leads to the initiation of LPO chain reactions resulting in the production in deleterious products which bind to the nucleophilic centers of DNA and proteins, resulting in significant cellular damage and impaired semen parameters [116,117]. Regarding Pb-induced testicular toxicity, various studies have shown that it is mediated by excessive production of ROS resulting in OS, evidenced by increased MDA levels, as well as depletion of antioxidant reserves [85,97,118,119]. Comparably, the findings of the present study revealed that rats exposed to PbAc exhibited decreased levels of the testicular enzymatic antioxidants CAT and SOD, and the non-enzymatic antioxidant GSH, as well as a rise in MDA levels, collectively indicating the generation of free radicals and consequent OS. On the other hand, HSD substantially restored the activity of CAT and the levels of GSH and reduced the increased MDA levels brought about by PbAc exposure. Collectively, the ameliorative effects of HSD observed in the current study might be attributed to the alleviation of oxidative damage induced by repro-toxic agents and regulation of the spermatogenesis process, as previously reported [88,120,121,122].

In addition to the direct effect on sperm cells and the generation of ROS, PbAc-induced testicular toxicity occurs via reduction in male reproductive hormones consequent to adverse effects on the HPT axis [85,118,123,124]. In the present study, a significant decrease in the levels of follicle-stimulating hormone (FSH), luteinizing hormone (LH), and testosterone was observed among PbAc-intoxicated animals. The reduction in the serum levels of testosterone is assigned to the decrease in LH known to regulate the production of sex steroids by Leydig cells [125,126]. It is noteworthy that the observed decrease in serum levels of LH and testosterone in PbAc-intoxicated rats might be responsible for the reduction in sperm counts, motility, and viability, as reported earlier [127]. In line with our findings, previous studies reported a reduction in serum FSH, LH, and testosterone levels in rats exposed to PbAc [37,128]. Nonetheless, a substantial increase in the serum levels of FSH, LH, and testosterone was observed in PbAc-intoxicated rats pre-treated with HSD. Knowing that OS can lower FSH, LH, and testosterone levels [129], the restoration of FSH, LH, and/or testosterone levels mediated via HSD might be attributed to its antioxidant effect as previously reported [88,122].

The current study revealed that the reduction in testicular weight following PbAc exposure in male Wistar rats was accompanied by histopathological alterations, represented mainly by vascular congestion; necrotic and degenerative modifications of seminiferous tubules; and ultimately, a disturbance in spermatogenesis. In support of such alterations, a substantial reduction in semen quality in PbAc-intoxicated rats was observed. Collectively, such findings are attributed to the reported PbAc-induced OS and hormonal imbalance. The observed testicular architectural changes are in line with earlier studies that reported comparable histopathological alterations [75,85,130]. On the other hand, pre-treatment of PbAc-intoxicated rats with HSD significantly improved the PbAc-induced histological alterations, which might be assigned to its antioxidant activity, as previously reported [88,122]. Similarly, it has been reported that HSD pre-treatment of rats intoxicated with bisphenol (BPA), an organic industrial compound that caused testicular tissue alterations comparable to PbAc, alleviated the testicular histopathological damage observed in BPA-intoxicated rats. It was observed that HSD administrated together with BPA resulted in minor edema, moderate hyperemia, and mild degeneration of spermatocytes [122]. These findings are in accordance with the mitigating effect of HSD against PbAc-induced testicular damage observed in the current study, where HSD pre-treatment showed a marked positive protective effect on testicular histology in PbAc-intoxicated rats, where both the integrity of spermatogoneal cells and active spermatogenesis were sustained and normal Leydig cells were observed.

## 5. Conclusions

The data obtained showed that exposure to PbAc induced testicular injury in male Wistar rats as demonstrated by impaired semen quality, altered biochemical parameters, and disrupted testicular histology. The reprotoxic effect of PbAc is mediated via induction of testicular oxidative stress, imbalance of sex hormones, and histopathological alterations. To our knowledge, our findings emphasize for the first time the plausible protective effect of the natural antioxidant HSD against PbAc-induced testicular injury that is attained mainly via restoration of cellular antioxidants and improvement in reproductive hormones. Hence, the current study paves the way for further studies exploring the plausible antioxidant therapeutic application of HSD for individuals exposed to lead poisoning.

## Figures and Tables

**Table 1:** Effect of HSD on spermatological parameters in PbAc-intoxicated male Wistar rats.

Groups	Count (×10^6^/mL)	Motility (%)	Viability (%)	Abnormality (%)
Ctrl	66.43 ± 3.29 *^a^*	54.21 ± 4.12 *^a^*	63.56 ± 3.91 *^a^*	11.14 ± 1.94 *^a^*
PbAc	29.05 ± 2.83 *^c^*	25.51 ± 5.02 *^c^*	23.53 ± 3.84 *^c^*	18.33 ± 2.06 *^b^*
HSD	65.16 ± 4.96 *^a^*	53.65 ± 3.78 *^a^*	59.44 ± 4.82 *^a^*	11.52 ± 2.73 *^a^*
HSD+PbAc	52.14 ± 3.23 *^b^*	46.37 ± 4.29 *^b^*	49.86 ± 3.79 *^b^*	12.26 ± 2.75 *^a^*

Data is expressed as mean ± SD. PbAc: lead acetate; HSD: hesperidin. Different small letters in the same column indicate significant difference at *p* ≤ 0.05.

**Table 2:** Effect of HSD on testicular antioxidants and oxidative stress biomarkers in PbAc-intoxicated male Wistar rats.

Groups	CAT	SOD	GSH	MDA
	(U/mg Protein)	(U/mg Protein)	(mmol/g Tissue)	(nmol/g Tissue)
Ctrl	2.74 ± 0.14 *^a^*	0.45 ± 0.04 *^a^*	23.86 ± 2.11 *^a^*	20.72 ± 1.87 *^c^*
PbAc	1.36 ± 0.19 *^c^*	0.26 ± 0.06 *^c^*	14.43 ± 2.24 *^b^*	38.59 ± 3.15 *^a^*
HSD	2.80 ± 0.25 *^a^*	0.41 ± 0.05 *^ab^*	21.94 ± 1.03 *^a^*	18.65 ± 2.32 *^c^*
HSD+PbAc	2.16 ± 0.18 *^b^*	0.35 ± 0.07 *^b^*	21.59 ± 0.8 *^a^*	27.44 ± 2.18 *^b^*

Data are expressed as mean ± SD. CAT: catalase; SOD: superoxide dismutase; GSH: reduced glutathione; MDA: malondialdehyde; PbAc: lead acetate; HSD: hesperidin. Different small letters in the same column indicate significant difference at *p* ≤ 0.05.

## Data Availability

All data analyzed during this study are included in this published article.
